# Identification and characterization of new Δ-17 fatty acid desaturases

**DOI:** 10.1007/s00253-012-4068-2

**Published:** 2012-05-27

**Authors:** Zhixiong Xue, Hongxian He, Dieter Hollerbach, Daniel J. Macool, Narendra S. Yadav, Hongxiang Zhang, Bogdan Szostek, Quinn Zhu

**Affiliations:** 1 Biochemical Science and Engineering, Central Research and Development, E. I. DuPont de Nemours, Experimental Station, Wilmington, DE 19880 USA; 2 Information and Computer Technology, E. I. DuPont de Nemours, Experimental Station, Wilmington, DE 19880 USA; 3 Corporate Center for Analytical Sciences, E. I. DuPont de Nemours, Experimental Station, Wilmington, DE 19880 USA

**Keywords:** ω-3 Desaturase, Δ-17 Desaturase, Long-chain polyunsaturated fatty acids, ω-3 Fatty acids, *Yarrowia lipolytica*, Metabolic engineering

## Abstract

ω-3 fatty acid desaturase is a key enzyme for the biosynthesis of ω-3 polyunsaturated fatty acids via the oxidative desaturase/elongase pathways. Here we report the identification of three ω-3 desaturases from oomycetes, *Pythium aphanidermatum*, *Phytophthora sojae*, and *Phytophthora ramorum*. These new ω-3 desaturases share 55 % identity at the amino acid level with the known Δ-17 desaturase of *Saprolegnia diclina*, and about 31 % identity with the bifunctional Δ-12/Δ-15 desaturase of *Fusarium monoliforme*. The three enzymes were expressed in either wild-type or codon optimized form in an engineered arachidonic acid producing strain of *Yarrowia lipolytica* to study their activity and substrate specificity. All three were able to convert the ω-6 arachidonic acid to the ω-3 eicosapentanoic acid, with a substrate conversion efficiency of 54–65 %. These enzymes have a broad ω-6 fatty acid substrate spectrum, including both C18 and C20 ω-6 fatty acids although they prefer the C20 substrates, and have strong Δ-17 desaturase activity but weaker Δ-15 desaturase activity. Thus, they belong to the Δ-17 desaturase class. Unlike the previously identified bifunctional Δ-12/Δ-15 desaturase from *F. monoliforme*, they lack Δ-12 desaturase activity. The newly identified Δ-17 desaturases could use fatty acids in both acyl-CoA and phospholipid fraction as substrates. The identification of these Δ-17 desaturases provides a set of powerful new tools for genetic engineering of microbes and plants to produce ω-3 fatty acids, such as eicosapentanoic acid and docosahexanoic acid, at high levels.

## Introduction

Long-chain polyunsaturated fatty acids (LCPUFAs), especially ω-3 LCPUFAs eicosapentanoic acid (EPA) and docosahexanoic acid (DHA), are essential nutrients critical to human nutrition and health. These fatty acids cannot be synthesized de novo in mammals and so must be obtained either directly through diet or indirectly through further desaturation and elongation of other polyunsaturated fatty acids (PUFAs) widely available in the diet, such as linoleic acid (LA) or α-linolenic acid (ALA) (Holman 1986; Lands 1992; Bézard et al. 1994; Moghadasian 2008). LCPUFAs are part of the cellular membrane system and play essential roles in determining the structure and function of the membrane (Rabinovich 1991; Stillwell and Wassall 2003; Ma et al. 2004). Physiologically, they are necessary for proper development in mammals, particularly in the developing infant brain, and for tissue formation and repair. They are also precursors to several important eicosanoids in mammals, e.g., prostacyclins, eicosanoids, leukotrienes, and prostaglandins (Funk 2001; Smith and Murphy 2002). Additionally, a high intake of long-chain ω-3 PUFAs produces cardiovascular protective effects (Dyerberg 1978; Shimokawa 2001; Kris-Etherton et al. 2002; von Schacky 2003; Calder 2004; Diniz et al. 2004; von Schacky and Harris 2007; Tavazzi et al. 2008). Numerous clinical studies have documented wide-ranging health benefits conferred by administration of ω-3 LCPUFAs against a variety of symptoms and diseases such as asthma, cancer, depression, diabetes, immune disorder, and skin conditions (Mickleborough et al. 2006; Colomer et al. 2007; Damsgaard et al. 2007; Amminger et al. 2010; Wall et al. 2010; Djoussé et al. 2011).

**Fig. 1:**
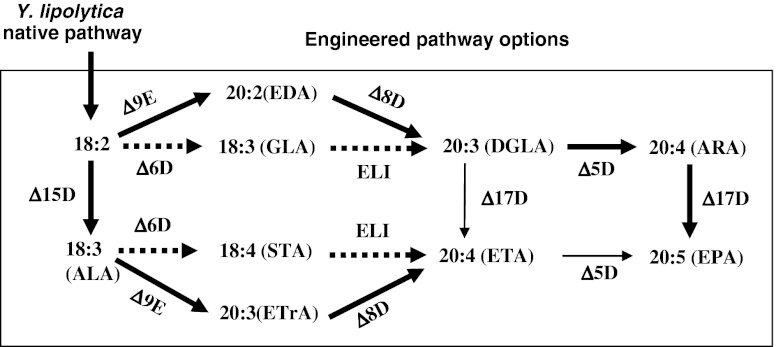
Schematic diagram of the fatty acid biosynthetic pathway leading to the production of EPA. *ALA* α-linolenic acid, *EDA* eicosadienoic acid, *GLA* γ-linolenic acid, *STA* stearidonic acid, *ETrA* eicosatrienoic acid, *DGLA* dihomo-γ-linolenic acid, *ETA* eicosatetraenoic acid, *ARA* arachidonic acid, *EPA* eicosapentaenoic acid, *Δ15D* Δ-15 desaturase, *Δ6D* Δ-6 desaturase, *ELI* Δ-6 elongase, *Δ9E* Δ-9 elongase, *Δ8D* Δ-8 desaturase, *Δ5D* Δ-5 desaturase, *Δ17D* Δ-17 desaturase

One of the main sources of EPA and DHA in humans is food containing these PUFAs. For example, cold water fish is an excellent source of both EPA and DHA (Chung et al. 2008; Baik et al. 2010). However, like mammals, these fish cannot synthesize EPA and DHA de novo, and instead obtain PUFAs directly or indirectly from their food. In contrast, microorganisms and phytoplanktons at the bottom of the food chain, such as diatoms, various types of algae, cyanobacteria, oomycetes, and fungi, can synthesize LCPUFAs de novo via one of two pathways, an anaerobic polyketide synthase pathway (Metz et al. 2001; Uttaro 2006) and an aerobic desaturase/elongase pathway (Sayanova and Napier 2004). The desaturase/elongase pathway can be further classified into the Δ-6 desaturase/Δ-6 elongase pathway and the Δ-9 elongase/Δ-8 desaturase pathway, as illustrated in Fig. 1 (see also Wallis et al. 2002; Damude et al. 2006; Zhu et al. 2010).

The Δ-6 desaturase/Δ-6 elongase pathway (called Δ-6 pathway from hereon) is predominantly found in algae, mosses, fungi, nematodes, etc., and is characterized by the production of γ-linolenic acid (GLA; 18:3 ω-6) and/or stearidonic acid (STA; 18:4 ω-3) as intermediates. This pathway can also produce EPA with the help of a Δ-15 desaturase, which converts LA to ALA followed by Δ-6 desaturase converting ALA to stearidonic acid (STA, 18:4 ω-3), then C18/20 elongase converting STA to eicosatetraenoic acid (ETA), and finally Δ-15 desaturase converting ETA to EPA.

The Δ-9 elongase/Δ-8 desaturase pathway (called the Δ8 pathway from hereon) operates in organisms such as euglenoid species (Wallis and Browse 1999). It is characterized by the production of eicosadienoic acid (EDA; 20:2 ω-6) and/or eicosatrienoic acid (ETrA; 20:3 ω-3) as intermediates. The Δ-8 pathway can also use ALA as an intermediate to produce EPA in combination with a Δ-15 desaturase, in which the Δ-15 desaturase converts LA to ALA, followed by Δ-9 elongase converting ALA to ETrA, then Δ-8 desaturase converting ETrA to ETA, and finally Δ-5 desaturase converting ETA to EPA. EPA can be further elongated by C20/22 elongase and desaturated by Δ-4 desaturase to form DHA.

As the world population expands, the ocean fishery resource is under increased pressure to supply food enriched in ω-3 fatty acids. It is generally recognized that the current practice of wild fish harvest and commercial fish farming are not sustainable over the long term. For example, to raise a kilogram of farmed salmon, oil from 4 kg of wild fish has to be consumed to produce the feed. Furthermore, pollution remains an issue with fish-derived ω-3 fatty acid oil. A sustainable supply of clean EPA and/or DHA could reduce the pressure on ocean resources and protect both the integrity of ω-3 fatty acid products and the environment. Toward this goal, a variety of different organisms including plants, algae, fungi, and yeast are being investigated as hosts for sustainable commercial production of EPA and DHA. Genetic engineering has demonstrated that the natural abilities of some hosts (even those natively limited to LA and ALA fatty acid production) can be substantially improved to produce important fatty acid molecules such as arachidonic acid (ARA), EPA, and DHA (Napier and Sayanova 2005; Graham et al. 2007; Zhu et al. 2010; Petriea et al. 2010; Tavares et al. 2011).

Genetic engineering of plants or microorganisms to produce EPA and DHA through the desaturase/elongase pathway requires the heterologous expression of various pathway enzymes. Regardless of whether the Δ-6 or Δ-8 pathway is used, a ω-3 desaturase is necessary for conversion of ω-6 PUFAs into their ω-3 counterparts. Because of the essential role Δ-17 desaturase enzymes play in the synthesis of long-chain ω-3 PUFAs, there has been considerable effort to identify and characterize these enzymes from various sources. However, so far only two Δ-17 desaturases have been isolated, one from *Saprolegnia diclina* (Pereira et al. 2004) and the other from *Phytophthora infestans* (GenBank accession no. CAJ30870). Identification of additional Δ-17 desaturases would be of great value to the effort of genetically engineering an organism to produce ω-3 fatty acids in a sustainable way. Here we report the identification and characterization of three genes encoding Δ-17 desaturases from the oomycetes, *Pythium aphanidermatum*, *Phytophthora sojae*, and *Phytophthora ramorum*. The enzymes were expressed in *Yarrowia lipolytica* to demonstrate that they are Δ-17 desaturases with high activity. We further compared their substrate specificity with the bifunctional Δ-12/Δ-15 desaturase from *Fusarium monoliforme*, another type of ω-3 desaturase (Damude et al. 2006).

## Materials and methods

### Strains

*Y. lipolytica* ATCC #20362 and ATCC #76982 (ade1 leu2-35 lyc1-5 xpr2) were purchased from the America Type Culture Collection. *Pythium aphanidermatum* was obtained from DuPont Agriculture Products (E.I. duPont de Nemours, Inc., Wilmington, DE, USA). Strain Y4070U is a ura3- derivatives of ATCC #20362 that produces ARA (Quinn Zhu, unpublished result), key features listed in Table 1. Strain Y8006U is a pex3::ura3 deletion strain containing the same set of key features as Y4070U. Strain L38 (leu2-35, lyc1-5, xpr2, YALi0B10153g deletion) is a derivative of ATCC #76982 in which the Δ-12 desaturase gene, YALi0B10153g, has been deleted (Zhang H and Yadav NS, unpublished result).

**Table 1:** Genetic elements introduced in Y4070U

Promoter	ORF	Enzyme activity	Terminator
GPD1	FmD12	*F. monoliforme*	PEX12
		Δ-12/Δ15 Desaturase	
EXP1	EgD9eS	*E. gracilis* Δ-9 elongase	LIP1
FBAINm	EgD9eS	*E. gracilis* Δ-9 elongase	LIP2
YAT1	ME3S	*M. alpina* C16 elongase	PEX16
FBAINm	EgD8M	*E. gracilis* Δ-8 desaturase	PEX20
EXP1	EgD8M	*E. gracilis* Δ-8 desaturase	PEX16
YAT1	FmD12	*F. monoliforme*	OCT
		Δ-12/Δ15 Desaturase	
GPAT	EgD9e	*E. gracilis* Δ-9 elongase	LIP2
EXP1	EgD5S	*E. gracilis* Δ-5 desaturase	PEX20
YAT1	RD5S	*Peridinuim* sp.	
		Δ-5 Desaturase	
FBAIm	EgD5	*E. gracilis* Δ-5 desaturase	ACO

EgD9eS is a codon optimized version of EgD9e, the *Euglena gracilis* Δ-9 elongase. ME3S is a codon optimized version of the *Mortierella alpina* C16 elongase. EgD8M is a codon optimized version of the *E. gracilis* Δ-8 desaturase. EgD5S is a codon optimized version of EgD5, the *E. gracilis* Δ-5 desaturase. RD5S is a codon optimized version of the *Peridinuim* Δ-5 desaturase. All promoter and terminator elements are from *Y. lipolytica*

### Media and chemicals

Yeast extract, yeast nitrogen base, and malt extract medium were from Difco Laboratories (Detroit, MI, USA). Trizol reagent was from Invitrogen (Carlsbad, CA, USA). Restriction enzymes were from Promega (Madison, WI, USA). Ex Taq polymerase was from TaKaRa Bio Inc. (Otsu, Shiga 520-2193, Japan). Qiagen miniprep DNA preparation kit and PCR purification kit were from Qiagen (Valencia, CA, USA). Universal Genome Walking kit was from BD Clontech (Mississauga, ON, Canada). Other chemicals were from Sigma Aldrich (St Louis, MO, USA). Yeast culture media were prepared according to standard recipe (Sherman 1991).

### Plasmids

**Fig. 2:**
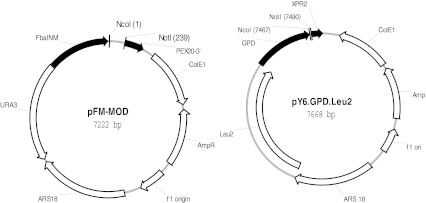
Maps of vector used to construct the D17 expression plasmids. Genetic elements present in the plasmids are as follows: ARS18, *Y. lipolytica* autonomous replication sequence; URA3, *Y. lipolytica* URA3 gene; LEU2, *Y. lipolytica* LEU2 gene; FbaINm, the *Y. lipolytica* FBA1Nm promoter; GPD, the *Y. lipolytica* GPD promoter; PEX20-3′, *Y. lipolytica* PEX20 terminator; XPR2, *Y. lipolytica* XPR2 terminator. The plasmids also contained the CoE1, f1 origin and ampR gene for propagation in *Escherichia coli*

Plasmids used in this study are derived from pFM-MOD and pY6GPDLEU2, both of which contained the ARS18 region of *Y. lipolytica* that allows the stable replication of the plasmid in the cell (Fournier et al. 1993). In addition, pFM-MOD also contained the *Y. lipolytica* URA3 marker, the FBAINm promoter (Hong et al. 2012) and the PEX20 terminator. pY6GPDLEU2 contained the *Y. lipolytica* LEU2 marker, GPD1 promoter (Hong et al. 2012) and XPR2 terminator (Fig. 2). The other plasmids are as follows: pFM-PaD17, a derivative of pFM-MOD containing the native *P. aphanidermatum* Δ-17 under the control of the FBA-In promoter; pFM-PaD17s, a derivative of pFM-MOD containing the synthetic codon optimized version of *P. aphanidermatum* Δ-17 ORF under the control of FBA-In promoter; pFM-PrD17s, a derivative of pFM-MOD containing the codon optimized version of *P. ramorum* Δ-17 ORF; pFM-PsD17s, a derivative of pFM-MOD, containing the codon optimized version of *P. sojae* Δ-17 ORF under the control of the FBA-In promoter; pY130GPDFmD15, a derivative of pY6GPDLEU2, containing the *F. monoliforme* Δ-12/Δ-15 ORF under the control of GPD promoter; pY138GPDPrD17, a derivative of pY6GPDLEU2 containing the codon optimized *P. ramorum* Δ-17 ORF under the control of GPD promoter; pY139GPDPsD17, a derivative of pY6GPDLEU2 containing the codon optimized *P. sojae* Δ-17 ORF under the control of GPD promoter; and pY140GPDPaD17, a derivative of pY6GPDLEU2 containing the codon optimized *P. aphanidermatum* Δ-17 ORF under the control of GPD promoter.

### DNA and RNA isolation

Total RNA and genomic DNA were isolated from *P. aphanidermatum* cells scraped off a malt extract agar plate, using the Trizol reagent (Invitrogen), according to the manufacturer’s protocol. Specifically, scraped cells were resuspended in 1 ml water and centrifuged for 30 s in an Eppendorf microfuge at 14,000 rpm. The cell pellet was resuspended in 0.75 ml Trizol reagent, mixed with 0.75 ml of 0.5 mm glass beads, and homogenized in a Biospec mini beadbeater (Bartlesville, OK, USA) at the highest setting for 3 min. The mixture was centrifuged in an Eppendorf centrifuge for 30 s at 14,000 rpm to remove debris and glass beads. The supernatant was extracted with 150 μl of 24:1 chloroform/isoamyl alcohol (Invitrogen). The upper aqueous phase was used for RNA isolation and the lower organic phase for DNA isolation, according to manufacturer’s protocol.

### cDNA synthesis

Double-stranded cDNA was synthesized directly from the *P. aphanidermatum* total RNA. Three microliters of total RNA sample (0.9 μg) was used as template for first strand cDNA synthesis using the BD-Clontech Creator^TM^ Smart^TM^ cDNA library kit following the manufacturer’s protocol. The resulting first strand cDNA synthesis mixture was then used as template for PCR amplification to generate double stranded cDNA, using the manufacturer’s condition. The PCR primers were 5′ PCR primer and CDSIII/3′ PCR primer from the kit. Amplification product was purified with a Qiagen PCR purification kit following the manufacturer’s protocol exactly. Purified cDNA product was eluted with 50 μl of water.

### Cloning of genomic and cDNA fragments containing the Δ-17 desaturase

Using the cDNA as template, a total of 49 different PCR amplification reactions were performed, using all possible combinations of the seven forward and seven reverse primers (Table 2) and the TaKaRa ExTaq 2× premix (Shiga, Japan). PCR products were cloned into pCR2.1-TOPO and sequenced. The Clontech Universal Genome Walker kit was used to obtain the 5′ and 3′ ends of the ORF, following the manufacturer’s protocol. For 5′ end, primers PaD-17-5-1 and DaD17-5-3 were used as gene-specific primers for the first and second round of PCR. For 3′ end, primers PaD17-3-1 and PaD17-3-2 were used (see Table 2 for primer sequences).

**Table 2:** Sequences of primers used to amplify potential Δ-17 desaturase

Primer	Nucleotide sequence	Amino acid sequence
PD17-F1	TTYTGGGGNTTYTTYACNGT	FWGFFTY
PD17-F2	TTCTTYACNGTNGGNCAYGA	FFTVGHD
PD17-F3	TTTTTYACNGTNGGNCAYGA	FFTVGHD
PD17-F4	ACNCAYCGNCAYCAYCAYAA	THRHHHK
PD17-F5	ACNCAYAGRCAYCAYCAYAA	THRHHHK
PD17-F6	AARAAYACNGGNAAYATYGA	KNTGNID
PD17-F7	AARAAYACNGGNAAYATAGA	KNTGNID
PD17-R1	TCRTCRTTRTGRTGNAGRAA	FLHHNDE
PD17-R2	TCRTCRTTRTGRTGYAARAA	FLHHNDE
PD17-R3	AARAARGCYTTDATDATNGG	PIIKAFF
PD17-R4	AARAAYGCYTTDATDATNGG	PIIKAFF
PD17-R5	TTRTGNGTNCCDATRTTATG	HNIGTHQ
PD17-R6	TTRTGNGTNCCDATRTTGTG	HNIGTHQ
PD17-R7	CCYTTNACRTANGTCCAYTC	EWTYVKG
PaD17-5-1	AATCTCGTCCTTGTCGATGTTG	
PaD17-5-3	TGAGGATGATCGAGTGAATGAG	
PaD17-3-1	CACCTACGTCTATGGCCTTAAG	
PaD17-3-2	CTGTACTACTTCGCCCCTCTCT	

The nucleic acid degeneracy code used are as follows: R = A/G, Y = C/T, D = A/G/T, and N = A/C/T/G

### GC analysis of fatty acid profile of *P. aphanidermatum*

Fatty acid profiles of *P. aphanidermatum* were obtained by GC analysis of extracted lipids from cells grown on malt extract agar plate (Difco Laboratories). Cells were scraped off the plate and resuspended in 600 μl of sodium methoxide dissolved in methanol. The sample was shaken for 20 min, and 50 μl of 1 M NaCl was added. After mixing, 600 μl of heptane was added. The sample was vortexed and centrifuged in an Eppendorf microfuge for 1 min. The upper layer was carefully separated from the lower layer and placed in a glass vial for GC analysis as previously described (Damude et al. 2006).

### Synthesis of the codon optimized *P. sojae* and *P. ramorum* Δ-17 desaturase ORFs

*P. sojae* and *P. ramorum* Δ-17 desaturases were identified by BLAST analysis using sequence of *P. aphanidermatum* Δ-17 as a query, against the *P. sojae* and *P. ramorum* databases of Department of Energy’s Joint Genome Program (http://genome.jgi-psf.org/Physo3/Physo3.home.html). The coding region of the Δ-17 enzymes were optimized based on the general rule of RNA stability, the codon preference, and the consensus sequence around AUG of genes from *Y. lipolytica*. DNA fragments encoding the codon optimized versions of candidate Δ-17 desaturase ORF were synthesized de novo by Genscript (Piscataway, NJ 08854, USA). A NcoI site was incorporated at the start codon and a NotI site after the stop codon to facilitate cloning of the synthetic ORFs into expression vectors. Internal NcoI sites were removed.

### Extraction and separation of lipid fractions

Acyl-CoAs and phospholipids from approximately 0.5 g of wet yeast pellets were extracted using a modified version of the method of Schjerling et al. and Domergue and Heinz et al. (Domergue et al. 2005; Schjerling et al. 1996). Yeast cultures were centrifuged to collect the cells. Wet cells were weighed into a 13-ml glass tube and resuspended in 800 μl of ice-cold water containing 1 ppm of 17:0 acyl-CoA as a standard. Three milliliters of 2:1 chloroform/methanol was then added and the cells vortexed five times for 1 min each, with intermittent cooling in an ice bath. One milliliter each of chloroform and water were added to each sample, and the mixtures were vortexed for one additional minute. After centrifuging for 5 min at 3,000 rpm, the upper phase was removed and discarded. The interphase containing the acyl-CoAs was treated with 400 μl of extraction buffer consisting of 2 ml 50 mM potassium phosphate buffer (pH 7.2, 2 ml IPA, 50 μl acetic acid, and 80 μl of 50 mg/ml BSA), 10 μl saturated ammonium sulfate, and 1.2 ml of 1:2 chloroform/methanol solution. After vortexing for 3 min, the samples were incubated fro 20 min at RT before being centrifuged at 3,000 rpm for 5 min. The supernatant was analyzed by HPLC for acyl-CoA, as described by Schjerling et al. (1996). The lower phase was transferred into a second 13-ml glass tube and dried using a turbovap. The dried down fraction was re-suspended in 6:1 chloroform/methanol and plated onto TLC plates. After developing with a solvent of 70:20:1 hexane/ethyl ether/acetic acid, the bands were scraped into 13-ml glass vials and esterified using 5 % methanolic HCl. They were then extracted with hexane and analyzed by GC as described below.

### GC analysis of fatty acid profile of *Y. lipolytica* transformants

Twenty-five-milliliter cultures of *Y. lipolytica* transformants in synthetic minimal medium (MM) (Sherman 1991) containing 2 % glucose were grown for 2 days at 30 °C and 250 rpm in 125-ml flasks. Cells in 10 ml of the culture were collected by centrifugation, resuspended in 25 ml of a medium containing 8 % glucose and 0.2 M sodium phosphate at pH 7.0 (HG medium), and allowed to grow for five more days at 30 °C and 250 rpm in 125 ml flasks. Cells were then harvested by centrifugation. Lipid extraction and fatty acid analysis were done as described previously (Damude et al. 2006). Substrate conversion efficiency for ω-3 desaturase is calculated as ${C_{\text{product}}}/\left( {{C_{\text{product}}} + {C_{\text{substrate}}}} \right).{C_{\text{product}}} = \%$ of product ω-3 fatty acid and *C*_substrate_ = % of substrate ω-6 fatty acid.

### ω-6 Fatty acid substrate feeding

Strain ATCC #76982 and L38 carrying various plasmids were grown in MM medium individually. Each overnight culture was diluted to an OD600 of 0.5 before aliquoting into three 3-ml cultures. After growth for another 6 h, the cultures were harvested by centrifugation and resuspended in fresh 3 ml MM medium containing 1 % Tergitol and 0.5 mM each of GLA, EDA, and ARA, and allowed to grow for 24 h, at which time they were harvested, washed once with 12 ml 0.5 % Triton X-100, and once with 12 ml distilled water. The pellets were analyzed for fatty acid composition (% of total fatty acids) by GC as described above.

### Sequence comparison and structure prediction

Sequence alignment was done with Vector NTI (Invitrogen) using the Clustal W software. Sequence homology was analyzed using the BLAST tools at NCBI. Initial prediction of transmembrane (TM) domain was carried out using program TMHMM (“Prediction of transmembrane helices in proteins”; TMHMM Server v. 2.0, Center for Biological Sequence Analysis, BioCentrum-DTU, Technical University of Denmark, DK-2800 Lyngby, Denmark). Multiple sequence alignment was generated for a number of Δ-17 desaturases and the three conserved His-rich motifs were identified. The topology model was adjusted to bring the three His-rich motifs to the cytoplasmic side (Diaz et al. 2002).

### GenBank accession numbers

*P. aphanidermatum* Δ-17 desaturase: FW362186.1

*P. ramorum* Δ-17 desaturase: FW362214.1

*P. sojae* Δ-17 desaturase: FW362213.1

## Results

### Fatty acid profile analysis of *P. aphanidermatum*

**Fig. 3:**
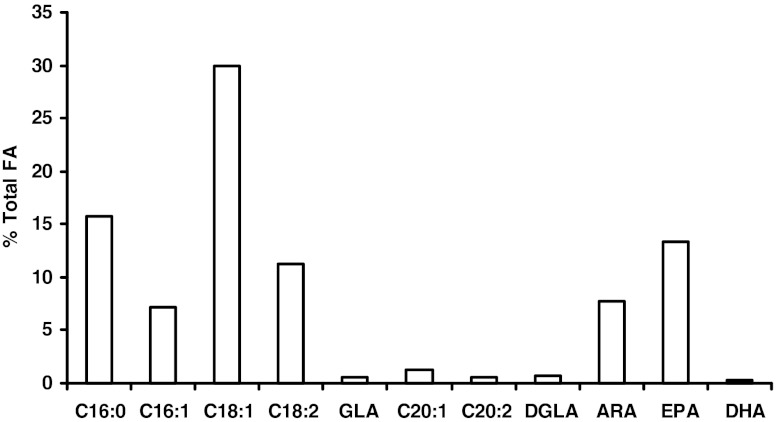
Fatty acid profile of *Pythium aphanidermatum. P. aphanidermatum* cells were grown on malt extract agar medium at room temperature for 3 days. Lipid extraction and GC analysis were done as described in “Materials and methods”

Oomycetes such as *Pythium* are known to produce EPA with ARA as one of the intermediates and thus should possess Δ-17 desaturase activity. One *P. aphanidermatum* strain previously isolated from turfgrass at DuPont Country Club in Wilmington, DE, was cultured on malt extract agar medium. GC analyses of lipids extracted from the cells showed that the strain produced significant levels of ARA and EPA, at 7.8 % and 13.5 % of total fatty acids respectively (Fig. 3). This suggests that the *P. aphanidermatum* strain has a complete synthetic pathway for EPA, including both a Δ-5 desaturase (capable of converting DGLA to ARA) and a Δ-17 desaturase (capable of converting ARA to EPA). Low amounts of GLA and EDA are also present. Thus, it is not possible to conclusively determine whether the organism possesses the Δ-6 or the Δ-8 pathway, or both, for EPA synthesis.

### Cloning of the Δ-17 desaturase cDNA from *P. aphanidermatum*

Based on the sequence of the two known Δ-17 desaturases from *S. diclina* and *P. infestans* in the public databases, we designed a set of 14 degenerated primers for amplification of the potential Δ-17 cDNA (see Table 2). The primers correspond to the highly conserved regions of the known Δ-17 desaturases. Genomic DNA and cDNA samples were prepared from *P. aphanidermatum* cells and used as template for degenerated PCR amplification of the Δ-17 gene. Three pairs of primers, PD17-F1/PD17-R5, PD17-F4/PD17-R2, and PD17-F6/PD17-R2, produced DNA fragments of the expected size, and these fragments were cloned and sequenced (see “Materials and methods” for details). Assembly of the sequences generated a 614-bp contig containing part of a putative ORF with 76 % identity to Δ-17 desaturase from *P. infestans* and 69 % identity to Δ-17 desaturase from *S. diclina*. DNA fragments corresponding to the 5′ and 3′ ends of the putative ORF were obtained by genome walking, and sequenced (see “Materials and methods”). Assembly of various sequences yielded a 1,533-bp contig that contained the entire ORF plus 5′ and 3′ untranslated regions. The coding region of the ORF from *P. aphanidermatum* (PaΔ17) is 1,080 bp long and encodes a peptide of 359 amino acids (GenBank accession number FW362186.1).

**Fig. 4:**
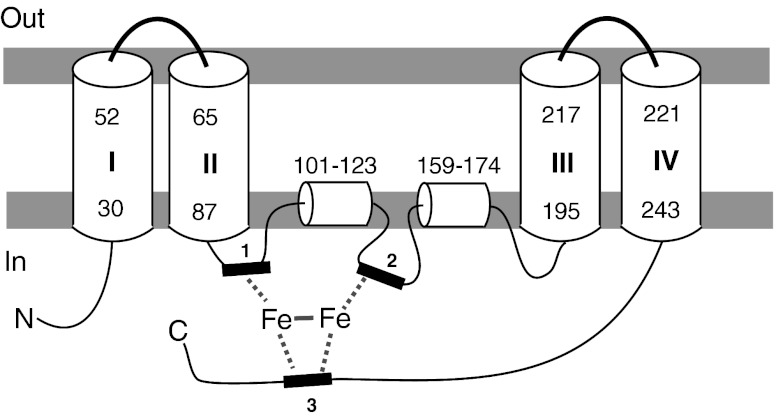
Predicted secondary structure of *P. aphanidermatum* Δ-17 desaturase. Initial prediction of transmembrane (TM) domain was carried out using program TMHMM. Multiple sequence alignment was generated for a number of Δ-17 desaturases and the three conserved His-rich motifs were easily identified. The topology model was adjusted to bring the three His-rich motifs to the cytoplasmic side

BLAST searches, using the sequence of the full-length ORF as the query against GenBank protein database, showed that it shared 76 % identity with the amino acid sequence of the Δ-17 desaturase from *P. infestans*, and 58 % with the Δ-17 desaturase from *S. diclina*. The results suggest that the cloned ORF encodes a ω-3 desaturase, likely a Δ-17 desaturase from *P. aphanidermatum*. Analyses of the sequence with structural prediction software indicate that this putative Δ-17 desaturase (PaD17) has four transmembrane domains and two intramembrane domains, as well as the typical histidine-rich desaturase motifs (Fig. 4). The C terminus of the protein contained a consensus ER membrane protein retention signal (KTKAN), suggesting that the protein is localized to the ER membrane (Jackson et al. 1990; Andersson et al. 1999).

### Identification of genes encoding Δ-17 desaturase from *P. sojae* and *P. ramorum*

**Fig. 5:**
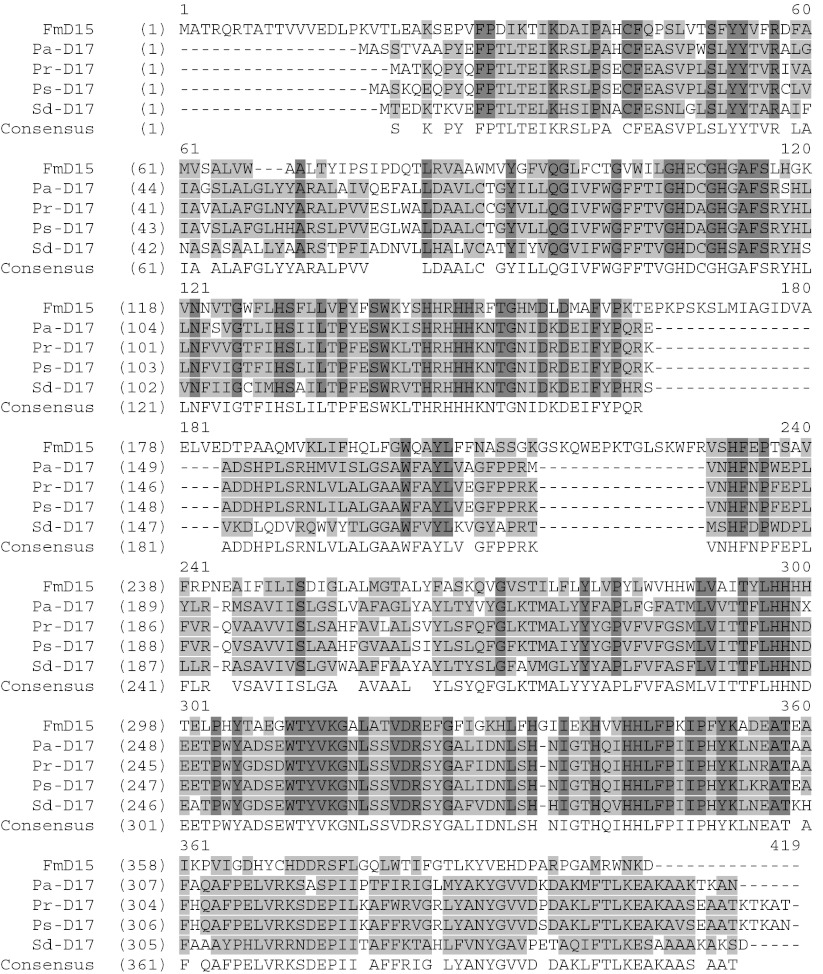
Sequence comparison of selected ω-3 desaturases. The amino acid sequences of five ω-3 desaturases were compared using the AlignX module of Vector NTI. FmD15: *F. monoliforme* Δ-12/Δ-15 desaturase. PrD17: *P. ramorum* Δ-17 desaturase. PsD17: *P. sojae* Δ-17 desaturase. PaD17: *P. aphanidermatum* Δ-17 desaturase. SdD17: *S. diclina* Δ-17 desaturase. *Lighter shaded areas* indicate conserved residues. *Darker shaded areas* indicate identical residues

To broaden the search for Δ-17 desaturases, we looked for this enzyme in other oomycetes species by using the amino acid sequence of PaD17 as a query to BLAST the *P. sojae* and *P. ramorum* databases of Department of Energy’s Joint Genome Program (http://genome.jgi-psf.org/Physo3/Physo3.home.html). In each organism, a candidate ORF for Δ-17 desaturase was found. As shown in Fig. 5, these ORFs share a high degree of homology to PaD17 as well as the Δ-17 desaturase from *S. diclina* (SdD17). Pair-wise comparison between and among the Δ-17 desaturase proteins from *S. diclina*, *P. infestans*, *P. sojae*, *P. ramorum*, and *P. aphanidermatum* using a Clustal W analysis showed that PaD17 is 74 % identical to Δ-17 from *P. ramorum* (PrD17), 73 % identical to Δ-17 from *P. sojae* (PsD17), and 59 % identical to SdΔ17. PsD17 and PrD17 are 91 % identical to each other and 58 % identical to SdD17. Each desaturase is also predicted to have the same set of membrane domains as that of PaD17, as well as the typical His-rich desaturase motifs and the ER membrane protein retention signal at the C terminus (see Figs. 4 and 5). The structure arrangements of all three Δ-17 desaturases are very similar.

### Confirmation of the activity of *P. aphanidermatum* Δ-17 desaturase

To confirm that the cloned PaD17 indeed has Δ-17 desaturase activity, we constructed an expression vector pFM-PAD17 containing the PaD17 ORF under the control of the strong constitutive *Y. lipolytica* promoter FBAIn (Hong et al. manuscript in preparation) from *Y. lipolytica* (see “Materials and methods” for details of the plasmid). The pFM-PAD17 construct was used to transform *Y. lipolytica* strain Y4070. This strain contained three copies of Δ-9 elongase genes, two copies of Δ-8 desaturase genes, and three copies of Δ-5 desaturase genes (“Materials and methods”), enabling it to produce ARA at approximately 10 % of total fatty acids. Introduction of a functional Δ-17 desaturase is expected to lead to production of EPA from ARA.

**Fig. 6:**
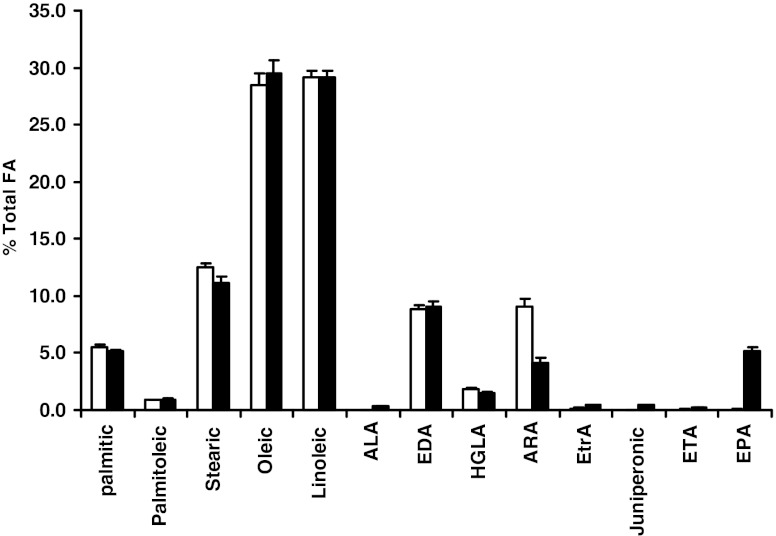
Fatty acid profile of Y4070 cells carrying pFM-MOD or pFm-PaD17. Y4070 cells carrying the designated plasmids were grown in SD medium followed by HG medium (see “Materials and methods” for details of the growth conditions). Lipid extraction and fatty acid profile analysis were done as described in “Materials and methods”

Various transformants were analyzed for their fatty acid profile as described in “Materials and methods”. As shown in Fig. 6, the parent strain Y4070 carrying the vector produced no detectable amount of EPA. In the Y4070 transformants carrying the pFM-PAD17 construct, EPA was produced at 5 % of the total fatty acid. The conversion efficiency of ARA to EPA is calculated to be 54 %. In addition to EPA, low concentrations of other omega-3 fatty acids, such as ALA, ETA, juniperonic acid, and ETrA, were also detected. The results confirmed that the PaD17 indeed encoded a Δ-17 desaturase capable of converting ω-6 ARA to ω-3 EPA.

### Improved expression and conversion efficiency of Δ-17 desaturase by codon optimization

Codon bias of an organism often leads to sub-optimal expression of a heterologous gene. We thus synthesized codon optimized versions of the Δ-17 desaturases from *P. aphanidermatum* (PaD17S), *P. sojae* (PsD17S), and *P. ramorum* (PrD17S) for enhanced expression in *Y. lipolytica* (“Materials and methods”). Comparison of the amino acid sequences of *P. sojae* Δ-17 and *P. ramorum* Δ-17 revealed that the N terminus of the PsD17 has two extra residues Q and E at position 5 and 6. To make a synthetic version of PsD17 with optimized codons (PsD17S), we designed the new gene without these two residues so that the N terminus region of PsD17S was similar to the PrD17S. In all, for PaD17S, 17.4 % of the 1,080-bp coding region were changed, which led to 48.6 % codons being optimized. The GC content was reduced from 61.8 % to 54.5 %. For PrD17S, 15.5 % of the 1,086-bp coding region was changed, leading to 44.2 % codons being optimized. The GC content was reduced from 64.4 % to 54.5 %. For PsD17S, 16.0 % of the 1,092-bp coding region was changed, leading to 46.2 % codons being optimized. The GC content was reduced from 65.1 % to 54.5 %.

The codon optimized versions of the three Δ-17 desaturases were used to replace the PaD17 ORF in plasmid pFM-PaD17 to generate pFM-PaD17S, pFM-PsD17s, and pFM-PrD17s. The three plasmids were then used to transform *Y. lipolytica* strain Y4070 individually. Transformants were analyzed for their fatty acid profiles. As shown in Table 3, all of the transformants produced significant levels of EPA, confirming the identity of the *P. ramorum* and *P. sojae* Δ-17 desaturases. The conversion efficiency of ARA to EPA with the codon optimized PaD17S improved from 54 % to 63.8 %. Thus, codon optimization was able to enhance substrate conversion of PaD17 by 14 %, presumably through improved translation efficiency. The conversion efficiency of PsD17S was about 65 % and that of PrD17S was about 60 %. The three Δ-17 desaturases have broadly similar activity levels and produced a similar fatty acid profile in strain Y4070.

**Table 3:** Conversion of ARA to EPA by the three synthetic versions of ω-3 desaturase

Sample	ARA (% of total)	EPA (% of total)	CE (%)
Y4070 + pFM-MOD	9.2	0	0
Y4070 + pFM-PaD17s	3.9	6.9	63.8
Y4070 + pFM-PrD17s	4.3	6.7	60.9
Y4070 + pFM-PsD17s	3.7	7.0	65.8

pFM-PaD17s carries the synthetic codon optimized version of *P. aphanidermatum* Δ-17, pFM-PrD17s carries the synthetic codon optimized version of *P. ramorum* Δ-17, and pFM-PsD17s carries the synthetic codon optimized version of *P. sojae* Δ-17. See “Materials and methods” for details of the plasmids

### Determination of omega-6 fatty acid substrate spectrum of different omega-3 desaturases

ω-3 Desaturases such as Δ-17 desaturase could possess either Δ-15 or Δ-17 desaturase activity, or both. We determined the Δ-15 desaturase activity of the three Δ-17 desaturases by transforming a wild-type *Y. lipolytica* strain, ATCC #76982, with expression constructs containing these ORFs under the control of the GPD1 promoter (see “Materials and methods”). As a comparison, the same vector carrying the previously reported Δ-12/Δ-15 bifunctional desaturase from *F. monoliforme* (Damude et al. 2006) was also used to transform the same strain. The fatty acid composition of the transformed strains with these constructs is shown in Table 4. Transformants carrying the desaturase ORFs all produced ALA. The one carrying the Δ-15 desaturase produced 29 % ALA, whereas those carrying the Δ-17 desaturases produced between 9 % and 12 % ALA. The conversion efficiency, expressed as concentration of product/(concentration of product + concentration of substrate), was thus 81.7 % for the FmΔ-12/Δ-15 desaturase and 24.6–34.6 % for the Δ-17 desaturases. PaD17s had the highest conversion efficiency among the Δ-17 s at 34.6 %. The results indicated that all three Δ-17 desaturases have significant Δ-15 desaturase activity, able to convert LA to ALA. As expected, the Δ-15 desaturase activity of *F. monoliforme* bifunctional Δ-12/Δ-15 desaturase was higher than all three Δ-17 desaturases.

**Table 4:** Average fatty acid composition of different transformants of wild-type (WT, ATCC #76932) and Δ-12-desaturase disrupted (d12KO) *Y. lipolytica* cells grown in glucose medium

		Fatty acid composition (% total fatty acid)			
Strain	Enzyme	C18:0	C18:1	C18:2	ALA
Wt	–	1.5	28.9	39.6	0
Wt	FmD15	2.1	33.7	6.5	29.1
Wt	PrD17s	1.6	30.4	29.1	9.5
Wt	PsD17s	1.5	30.8	26.5	11.8
Wt	PaD17s	1.7	33.6	23.1	12.2
D12 KO	-	2.1	71.4	0	0
D12 KO	FmD15	2.5	55.0	0.6	15.7
D12 KO	PrD17s	2.2	69.5	0	0
D12 KO	PsD17s	2.1	70.2	0	0
D12 KO	PaD17s	2.6	69.5	0	0

Cells were grown in 3 ml MM culture for 2 days in triplicate. Fatty acid analysis was done as described in “Materials and methods”

The FmΔ-12/Δ-15 desaturase is a bi-functional desaturase with high level of ω-6 desaturase activity as well. To test if the Δ-17 desaturases were also bi-functional desaturases, the Δ-12 desaturase activity of the three Δ-17 desaturases was determined by transforming their expression constructs into a Δ-12 desaturase-disrupted *Yarrowia lipolytica* strain L38, which was derived from ATCC #76982. The FmΔ-12/Δ-15 desaturase was used as a control. If there is any Δ-12 desaturase activity present, we expect to see the production of LA and ALA in these transformants. The fatty acid composition of L38 with different constructs (Table 4) revealed that cells carrying the three Δ-17 desaturases did not produce detectable levels of LA or ALA, indicating a lack of any Δ-12 desaturase activity. In contrast, cells carrying the *F. monoliforme* bifunctional Δ-12/Δ-15 desaturase produced a significant level of ALA (due to the high Δ-15 activity) and residue level of LA, as expected. Thus, unlike the Δ-12/Δ-15 desaturase, the newly identified Δ-17 desaturases are not dual function desaturases, but strictly ω-3 desaturases.

**Fig. 7:**
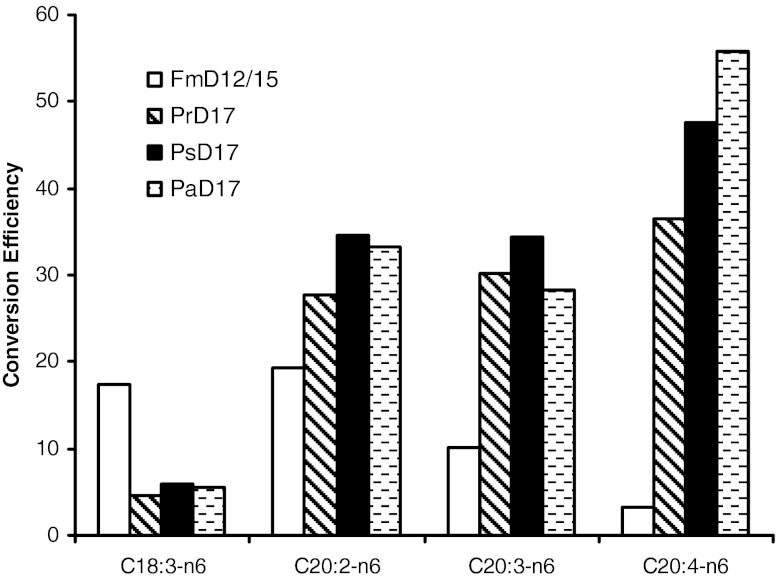
Conversion efficiency of ω-3 desaturases on different fatty acids produced in the engineered strain. Strain L38 carrying different Δ-17 plasmids was grown in the presence of a mixture of 0.5 mM each of exogenous GLA, EDA, and ARA. Cells were harvested and fatty acid content analyzed as described in “Materials and methods”. Conversion efficiency is calculated as ${1}00\% \times {C_{\text{product}}}/\left( {{C_{\text{product}}} + {C_{\text{substrate}}}} \right)$, where *C*_product_ and *C*_substrate_ are concentrations of the product and substrate of the Δ-17 desaturase

Finally, we tested the relative activity of the three different Δ-17 desaturases on ω-6 fatty acids other than LA. L38 cells carrying different Δ-17 plasmids were grown in the presence of a mixture of 0.5 mM each of exogenous GLA, EDA, and ARA. The calculated conversion efficiency toward each ω-6 fatty acid, based on the fatty acid composition of the cells carrying different enzymes, revealed the relative activity of the enzymes toward these substrates. Figure 7 shows the calculated conversion efficiency of each enzyme toward different ω-6 substrates. The three Δ-17 desaturases have relatively high conversion efficiency with C20 ω-6 substrates, but lower conversion efficiency with GLA, a C18 ω-6 substrate. This is different from the Δ-12/Δ-15 desaturase, which has high activity toward GLA and C20:2 ω-6. Thus, the three Δ-17 desaturases appeared to be omega-3 desaturases that preferred C20 substrates. The PaD17s had the highest conversion efficiency toward ARA (C20:4).

### Fatty acid profile in various lipid fractions

It has been reported that although most desaturases use fatty acid moiety in phospholipid (PL) as substrate, some could use acyl-CoA as substrate (Pereira et al. 2004; Domergue et al. 2005). We thus analyzed the fatty acid profile of phospholipid (PL), acyl-CoA, and triglyceride (TAG) fractions to determine if the Δ-17 desaturase prefer PL or acyl-CoA. Wt *Y. lipolytica* strain ATCC20362 carrying pFM-PrD17 was grown in SD medium overnight. ARA (1 mM) was added to the medium and samples were taken at different time points. Lipids from each sample were extracted, separated, and analyzed for their fatty acid profile. At 5 min time point, there is no ARA or EPA in the PL fraction, but ARA is present in free fatty acid fraction. By HPLC–mass spectrometry, we could detect a small amount of EPA in the CoA fraction, suggesting that the Δ-17 desaturase could use Acyl-CoA directly. However, the amount of EPA is extremely small, at less than 0.1 % of the ARA amount (data not shown). Since ARA is not incorporated into the PL fraction (Pereira et al. 2004), it is difficult to determine if ARA in PL fraction can be used as a substrate. We thus analyzed the fatty acid composition of PL and total lipid in an engineered ARA producing strain carrying pFM-PrD17. Strain Y8006 carrying pFM-PrD17, grown in either SD medium or HG medium, were analyzed for fatty acid distribution of PL and total lipid as described in “Materials and methods”. As shown in Table 5, in HG medium the PL fraction contained a higher percentage of EPA than the total lipid fraction. In SD medium, under normal growth condition, PL fraction contained slightly less EPA than total lipid fraction. This suggests that the Δ-17 desaturase could use PL as a substrate. Furthermore, the enzyme appears to prefer PL as a substrate, at least under oleaginous conditions, since the conversion efficiency is much higher in PL fraction than CoA fraction in HG medium.

**Table 5:** ARA and EPA content in PL, CoA, and total lipid fractions

Sample	ARA (% of total)	EPA (% of total)	CE (%)
SD-PL	5.0	2.3	31.6
SD-CoA	6.6	3.5	34.3
SD-Total	3.4^a^	2.1^a^	37.9
HG-PL	5.8	6.3	52.1
HG-CoA	4.7^a^	1.6^a^	25.7
HG-Total	3.7	2.9	43.6

For SD sample, cells were grown in 100 ml SD medium for 2 days. For HG sample, cells were grown in 100 ml SD medium for 2 days, followed by 3 days in 100 ml HG medium. Lipid extraction and fatty acid analysis were done as described in “Materials and methods”

^a^In CoA sample, ARA and EPA amount is relative peak area rather than % total

## Discussion

Oomycetes such as *Pythium* and *Phytophtora* are known to produce LCPUFAs, such as ARA and EPA (Stinson et al. 1991). Fatty acid profiles of these organisms suggest that they produce these LCPUFAs through the desaturase/elongase pathway, rather than the polyketide pathway used by marine bacteria and thraustochytrids (Stredansky et al. 2000). These organisms thus are excellent sources for various desaturase and elongase genes of the LCPUFA pathway. We have identified and isolated three new ω-3 desaturases from these organisms that convert ω-6 fatty acids to the ω-3 unsaturated form. The newly identified ω-3 desaturases belong to the so-called type II desaturase that introduces double bond near the methyl end of an unsaturated fatty acid (Uttaro 2006). They are highly similar in amino acid sequences, with >73 % identity. Each contained the signature desaturase motifs, six membrane domains, and share extensive similarity in secondary structure arrangement. However, they share less identity to other ω-3 desaturases such as the Δ-17 desaturase from *S. diclina* (55 % identity) and the Δ-15 desaturase from *F. monoliforme* (31 % identity). Nevertheless, the secondary structure arrangements were similar among these ω-3 desaturases, based on structure predictions. All three desaturases also contained the consensus ER membrane protein retention signal at the C terminus, suggesting that they are localized to the ER membrane like that of the *S. diclina* Δ-17 desaturase (Pereira et al. 2004). Similar to the *S. diclina* Δ-17 and other ω-3 desaturases, they lack the cytochrome b5 domain and need to interact with independent cytochrome b5 to be active (Pereira et al. 2004).

The activity and substrate spectrum of these enzymes are analyzed by expression of the genes in both wild-type and genetically engineered *Y. lipolytica* strains. The wild-type *P. aphanidermatum* enzyme, when introduced into the ARA producing strain Y4070, converted 54 % of the endogenous ARA to EPA. The codon optimized version converted 10 % more ARA than the wild-type version (64 % versus 54 %). The codon optimized version of *P. sojae* and *P. ramorum* enzymes also converted >60 % of ARA to EPA. This result showed that these enzymes are highly active when expressed in *Yarrowia*. It suggests that they retained the correct conformation and intracellular location. The enhanced activity of the codon optimized version of *P. aphanidermatum* enzyme, compared to the wild type, indicated a further improvement in expression level resulting from codon optimization.

Like Δ-17 desaturases from *S. diclina*, the three new Δ-17 desaturases could also convert different types of ω-6 fatty acids to ω-3 fatty acids. Fatty acid feeding experiments showed that these three ω-3 desaturases had the strongest substrate preference for ARA, relatively lower preference for EDA and DGLA, and least preference for GLA (Fig. 7). The activity of the three enzymes toward C18 substrate GLA is in contrast to that of the *S. diclina* Δ-17 desaturase, which was reported to be an exclusive C20 desaturase with no activity on the C18 substrates (Pereira et al 2004). These three ω-3 desaturases had also significant levels of Δ-15 desaturase activity, as shown by their ability to convert LA to ALA. The conversion efficiency of the Δ-15 activity of these enzymes is about the same as the Δ-17 conversion efficiency with substrates other than ARA, such as EDA and DGLA. Thus, it appears that the enzymes are optimized for the production of EPA but have broad substrate specificity at lower activity levels. Furthermore, the substrate specificity analysis showed that these enzymes are the v + z-type desaturases that require a pre-existing double bond in the substrate molecule, and generate a new double bond three carbon away towards the methyl end of the fatty acids (Shanklin and Cahoon 1998). Expression of these three ω-3 desaturases in a Δ-12 deletion strain, L138, showed that, unlike the previously reported bi-functional Fm Δ-12/Δ-15 desaturase, none of them have any detectable Δ-12 desaturase activity. Therefore, the enzymes are not dual function desaturases like the *Fusarium* Δ-12/Δ-15 desaturase (Damude et al. 2006). The sequence divergence between the *Pythium* enzymes, the *S. diclina* enzyme and the *F. monoliforme* enzyme probably contributed to the observed difference in substrate specificity. As shown in Fig. 5, the *F. monoliforme* enzyme in particular has two stretches of extra amino acids (a.a. 165–184 and 214–230) that are not present in other Δ-17 desaturases. This could be the key factor determining the substrate specificity of the Δ-15 enzyme. More detailed mutagenic study will have to be done to fully understand the factors contributing to the substrate specificity.

Our analysis on the substrate preference of Pr Δ-17 showed that a small amount of activity on acyl-CoA substrate can be detected when cells are fed with ARA supplied in the culture medium. However, compare with the total activity, this acyl-CoA based activity is insignificant. The *S. diclina* Δ-17 desaturase was reported to desaturate fatty acids not connected to PL (Pereira et al. 2004). It was not established whether Sd Δ-17 also acts on PL because ARA supplied in the medium is not incorporated into PL (Pereira et al. 2004). Taking advantage of the availability of strains that produce ARA de novo, we were able to show that in an engineered ARA producing strain carrying Pr Δ-17, the PL fraction contained higher percentage of EPA than either the CoA fraction, or the total lipid fraction under oleaginous condition. This strongly suggests that these Δ-17 desaturases could use, and likely prefer, fatty acids in PL fraction as substrates. It also suggests that like the Sd Δ-17 desaturase, these enzymes do have activity toward CoA substrates as well.

The high activity of these newly identified ω-3 desaturases render them excellent choices for genetic engineering of organisms for the production of EPA. One of the major issues in engineering an organism for efficient production of omega-3 fatty acids is the conversion efficiency of the enzymes used for engineering. With the elongase/desaturase pathway, the precursors of the ω-3 fatty acids are ω-6 fatty acids. The physiological effects of the ω-3 and ω-6 fatty acids are quite different (Simopoulos 2011; Ramsden 2010). It is thus important to control the ω-6 fatty acid at a low level in the final product. To do this, the ω-3 desaturase activity level in the host needs to be high. The previously characterized *S. diclina* Δ-17 desaturase, when expressed in yeast, has a conversion efficiency of only 26 % in a feeding experiment. The Δ-17 enzymes described in this study have conversion efficiencies ranging from 36 % to 56 % in feeding study, significantly higher than that of the *S. diclina* enzyme. The activity is even higher toward endogenous ω-6 fatty acids. We have demonstrated that in a genetically engineered ARA producing *Y. lipolytica* strain, the expression of any of the PaD17, PrD17, and PsD17 from a low copy plasmid led to the conversion of up to 64 % of the ARA to EPA. Importantly the codon optimized version produced more EPA, suggesting that it is possible to increase the conversion efficiency via enhanced expression of the enzyme. By constructing a stable transformant with three copies of Δ-17 desaturase gene integrated into the genome, the conversion efficiency from ARA to EPA could be as high as 95 % (Zhu et al., unpublished result). Thus, by using multiple copies of the highly active Δ-17 desaturase, it is possible to lower the ω-6 fatty acid concentration to less than 5 %. Having multiple Δ-17 desaturases allowed us to avoid repeated use of a single ORF, thereby reducing the chance of unwanted recombination events and increasing the stability of the engineered strain. This paves the way for the construction of production organisms that are capable of producing high levels of ω-3 fatty acids EPA and DHA.
